# Intra-articular Delivery of Recombinant Interleukin-1 Receptor Antagonist Protein (Anakinra) Enhances Graft Function in a Porcine Model of Osteochondral Repair

**DOI:** 10.1177/03635465251401225

**Published:** 2026-01-21

**Authors:** Brendan D. Stoeckl, Rachel A. Flaugh, Akbar N. Syed, Kendall M. Masada, Elizabeth R. Bernstein, Elisabeth A. Lemmon, Austin C. Jenk, Lorielle G. Laforest, Natalie L. Fogarty, Bijan Dehghani, Carla R. Scanzello, James L. Carey, David R. Steinberg, Robert L. Mauck

**Affiliations:** * Department of Orthopaedic Surgery, University of Pennsylvania, Philadelphia, Pennsylvania, USA; † Department of Bioengineering, University of Pennsylvania, Philadelphia, Pennsylvania, USA; ‡ Translational Musculoskeletal Research Center, CMC Veterans Administration Medical Center, Philadelphia, Pennsylvania, USA; § CReATE Motion Center, CMC Veterans Administration Medical Center, Philadelphia, Pennsylvania, USA; ‖ Children’s Hospital of Philadelphia, Philadelphia, Pennsylvania, USA; ¶ Division of Rheumatology, University of Pennsylvania, Philadelphia, Pennsylvania, USA; Investigation was performed at University of Pennsylvania and the CMC VA Medical Center, Philadelphia, Pennsylvania, USA

**Keywords:** articular cartilage resurfacing, biologic healing enhancement, biomechanics articular cartilage, clinical medicine by anatomic region, clinical medicine by specialty interest, growth factors/healing enhancement, knee, general, research, in vivo or in vitro

## Abstract

**Background:**

Osteochondral autografts may be subject to suboptimal healing and graft degeneration due to surgical insult and the inflammatory environment of an injured joint.

**Purpose/Hypothesis:**

The purpose of this study was to alleviate the negative effect of this inflammatory milieu on the healing of osteochondral grafts by treating operative joints with interleukin-1 receptor antagonist (IL-1ra; Anakinra) in a porcine model. It was hypothesized that such treatment would reduce markers of inflammation and lead to improved implant structural and functional outcomes.

**Study Design:**

Controlled laboratory study.

**Methods:**

The authors performed an osteochondral autograft transfer (OAT) procedure on the weightbearing surface of the medial femoral condyle of adult Yucatan minipigs. Beginning 1 week after surgery, a subset of animals received an intra-articular injection of 8 mg Anakinra in the operative stifle on a weekly basis for 4 weeks. At the 5-week endpoint, mechanical testing of the cartilage was performed, synovium and osteochondral specimens were analyzed histologically using semiquantitative scoring systems, and subchondral bone was analyzed via micro–computed tomography.

**Results:**

IL-1ra–treated joints showed significantly less histological evidence of synovial inflammation. Autografts from treated joints showed better retention of mechanical properties and better histological scores.

**Conclusion:**

Results indicate that intra-articular IL-1ra administration after surgery significantly improves graft structure and function and dramatically enhances healing.

**Clinical Relevance:**

This study demonstrates that local provision of adjuvant anti-inflammatory therapeutics after OAT may enhance healing and protect graft integrity. This not only has implications for current clinical practice of osteochondral autograft (and allograft) procedures but also may allow expanded indications for advanced biological repair in a greater number of patients.

Articular cartilage exhibits a poor endogenous healing response due to its avascular and hypocellular nature, and any damage that articular cartilage sustains may progress to the joint-wide disease of osteoarthritis (OA).^18^ Knee OA in particular represents an enormous clinical burden, with treatment for end-stage disease limited to partial or total joint replacement (TJR).^11^ While limiting pain and restoring function, these metal and plastic devices begin to wear immediately on implantation, and many eventually require costly and invasive revision surgeries.^1^ TJR of the knee carries elevated risk in younger patients given the limited functional longevity of the artificial joint.^1^

Given this, earlier treatment through biological cartilage reconstruction with living tissue may be an optimal solution, and several such interventions exist for early-stage cartilage damage. These include marrow-stimulating techniques such as microfracture^5,16^ and more advanced multistage procedures such as matrix-associated autologous chondrocyte implantation, in which a patient's healthy cartilage cells are harvested, seeded in a collagen matrix, and reimplanted into the defect.^62^ However, these procedures are costly and involve a long postoperative recovery period, and the repair tissue that is formed is inferior to native cartilage.^17^ Osteochondral autografting (or allografting), which involves moving an osteochondral unit from a nonweightbearing portion of the knee (or from a fresh cadaver) into a defect site, is an attractive alternative to these cell-based procedures. This process restores a defect with immediately viable and mechanically robust cartilage tissue,^21^ has excellent outcomes, and can return patients to full weightbearing faster than other repair procedures.^8,59^ However, these implants suffer from poor cartilage integration, may also suffer from poor bony integration, require precise shape and size matching, and, in the case of allografts, require strict disease screening^18^ and storage considerations.^3^ Moreover, like many biological repair strategies, these procedures are only indicated for use in otherwise healthy knees.^17^

Several groups have investigated osteochondral grafting in large animal models, both to expand the basic science knowledge of parameters impacting graft function^38,43-45^ and to repair naturally occurring defects in companion animals^10^ and zoological specimens.^23^ In these studies, close inspection of grafted tissue after implantation has revealed several limitations. For instance, Kleemann et al^28^ performed an osteochondral autograft procedure on the weightbearing surface of femoral condyles in an ovine model and found reduction in cartilage mechanical properties 3 months after implantation, as well as abnormally fibrillated graft edges in both autograft and adjacent tissue. Similarly, in a caprine model of an osteochondral autograft transfer (OAT) procedure, Raub et al^49^ found significant degenerative structural changes in the graft tissue. While observing healing of subchondral bone, Tibesku et al^58^ also observed poorly healed and degenerated cartilage after an OAT procedure in sheep. And in a porcine model, osteochondral autografts show poor cartilage integration with the surrounding tissue.^22^

OAT procedures, even in the most ideal surgical scenario, are inherently traumatic to cartilage and other joint tissues. Despite the excellent primary stability that press-fitting of grafts provides,^13^ the forces involved in graft placement may be deleterious to the health of the cartilage.^25,61^ Not only does the osteochondral graft undergo potentially damaging loading during the process of implantation, but also initial surgical insult likely produces a joint-wide inflammatory response. Inflammation is one of the critical drivers of joint pathology after joint injury^19^ and a major driver of chronic pain and progression in OA.^35,41,50^ Active inflammation can also inhibit the repair process and limit the efficacy of biological restoration strategies.^15,20,36,39^ Catabolic cytokines and matrix metalloproteinases (MMPs) present in an inflamed joint degrade native extracellular matrix (ECM) and can prevent normal chondrogenic differentiation.^60^

Several molecules with potentially disease-modifying properties have been explored for the treatment of inflammation associated with joint injury.^6,24,27^ One cytokine to target, interleukin-1β (IL-1β),^14^ is associated with both chronic and acute joint injury.^2^ IL-1β is upregulated in osteoarthritic knees^35,50^ and in the synovial fluid of human patients after osteochondral allograft procedures.^4^ Interleukin-1 receptor antagonist (IL-1Ra) competes with IL-1β for binding sites, blocking downstream signaling.^27^ Anakinra, a recombinant IL1-Ra, has been approved by the Food and Drug Administration for the treatment of rheumatoid arthritis and autoinflammatory syndromes and is often used off-label for the treatment of inflammation in gout flares.^51^ It has been investigated for the treatment of inflammation associated with OA.^27^ In a canine model of surgically induced cruciate ligament rupture, Anakinra treatment reduced macroscopic and histological evidence of OA progression in the knee at an early (4-week) time point,^7^ and in a human trial, Anakinra administration after an acute anterior cruciate ligament injury significantly improved the Knee injury and Osteoarthritis Outcome Score compared with placebo.^29^

Given these findings, in this study, we examined the effects of adjuvant therapy (intra-articular injection of recombinant IL1-Ra [Anakinra]) after an OAT procedure on the weightbearing surface of the medial femoral condyle in the porcine stifle joint. Our goal was to determine whether such adjuvant therapies could improve the function of the graft tissue and ameliorate inflammatory changes to the operated joint overall. To assess this, we investigated synovial pathology, cartilage mechanics, subchondral bone healing, and osteochondral histopathology 5 weeks after surgery, both with and without Anakinra treatment.

## Methods

### Surgical Methods and Joint Injections

**Figure 1.**
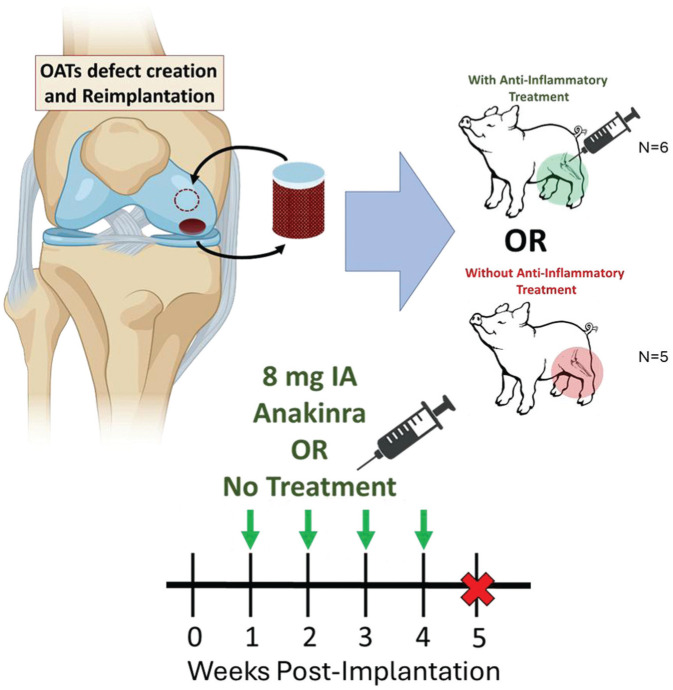
Experimental design. All animals underwent a unilateral osteochondral autograft transfer (OAT) procedure on the medial femoral condyle. A 7 mm–diameter by approximately 10 mm–height donor plug was harvested and press-fit into a 6 mm–diameter by approximately 10 mm–deep recipient defect. The donor site was left empty. The relative positions of each site distal or proximal on the medial femoral condyle were alternated between cases. For the Anakinra group, beginning 1 week after surgery, sedated animals received a weekly intra-articular injection of 8 mg Anakinra in a 1-mL saline carrier into the operative stifle. All animals were sacrificed 5 weeks after surgery. Image partially created using BioRender.

This study received approval from the Institutional Animal Care and Use Committee at the University of Pennsylvania. Surgery was performed unilaterally in 11 skeletally mature (12-month-old) Yucatan minipigs. A medial parapatellar arthrotomy was followed by patellar subluxation and hyperflexion of the stifle to allow for visualization of the medial femoral condyle. Next, a 6 mm–diameter × 10 mm–depth defect was created on the medial femoral condyle using a standard Arthrex OAT kit. A second defect, 7 mm in diameter × 10 mm in depth, was created, and the resulting osteochondral plug was press-fit into the first defect. Efforts were made to ensure congruence between the cartilage surfaces of the defect site and recipient plug, matching both surface curvature and defect depth. The empty 7-mm donor site served as a negative empty control. Each defect was made on the weightbearing midline of the medial femoral condyle, and their relative positions proximal or distal were alternated between subjects. Animals were singly housed on nonslip mats postoperatively, allowed unrestricted weightbearing as tolerated, and monitored daily for signs of pain and discomfort by laboratory animal veterinarians, with analgesia given as needed. Postoperative lameness resolved in all animals by the study endpoint. In a subset (n = 6) of animals, beginning 1 week after surgery, 8 mg Anakinra was injected intra-articularly in a saline carrier (1 mL) into the operative stifles of sedated animals on a weekly basis for 4 weeks. Animals were sacrificed 5 weeks after surgery (Figure 1).

### Synovial Histopathology

After sacrifice, both operative and contralateral stifle joints were dissected, and synovial tissue was isolated from the joint lining along the distal edge of the patella. Synovium was then fixed in neutral buffered formalin, paraffin processed, and sectioned (to 7-micron thickness). Tissues were then stained with hematoxylin and eosin and imaged at ×10 using a Zeiss Axio Scan.Z1 slide scanner. Four fields of view from each slide were scored by 4 blinded observers (B.D.S., R.A.F., E.R.B., L.G.L.) for synovial pathology with parameters of lining hyperplasia, fibrosis, inflammation, and vasculature according to a modified version of the Osteoarthritis Research Society International (OARSI) histopathology scoring system.^34,52^

### Cartilage Mechanics

Osteochondral segments of the central portion of each medial femoral condyle were isolated. Specimens were potted using Field metal—a low melting temperature alloy containing bismuth, indium, and tin—with the cartilage surface facing up. A custom indention setup in conjunction with an Instron 5948 electromechanical test frame was used to apply a creep load of 0.1 N with a 2 mm–diameter spherical indenter. The load was held constant for 900 seconds while displacement was recorded. Specimens were tested in a phosphate-buffered saline bath at room temperature. Indentation creep tests were performed in the center of each autograft or empty defect as well as 5 mm adjacent to the edge of each defect. Contralateral medial femoral condyles were tested similarly at a location corresponding to the autograft location in the operative stifle. Resulting deformation curves were fitted to a Hertzian biphasic analytical model, and material parameters of compressive modulus (*E_y_*_–_), tensile modulus (*E_y_*_+_), and zero-strain permeability (*k*_0_) were computed.^40^

### Micro–Computed Tomography

To assess bony features, after mechanical testing, each osteochondral specimen was fixed in 10% neutral buffered formalin and then scanned via micro–computed tomography (microCT; Scanco µCT 50) at 70 kVp, 85 µA, with a voxel size of 10.3 µm. DICOM files were imported into Dragonfly analysis software (Comet Group). Each empty defect and autograft repair was assigned a standardized cylindrical region of interest (ROI) 4 mm in diameter and 5 mm in height oriented parallel to the long axis of the defect and 1 mm below the surface of the bone-cartilage interface (see Figure 6, A and B). The built-in bone analysis tool in Dragonfly was used to compute bone volume fraction in each of these ROIs. After the initial scan was obtained, specimens were immersed in Lugol solution (10% KI, 5% I_2_ in water) for 24 hours to increase the radiopacity of the cartilage. Specimens were then rescanned via microCT with the same parameters as the first scan.

### Osteochondral Histology

Medial femoral condyle osteochondral specimens were decalcified by immersion in Formical-2000 for 5 weeks with weekly solution changes, and the paraffin was processed. Specimens were embedded such that the sectioning plane was oriented sagittally. Ten-micron sections were cut through the width of each autograft and empty defect. Sections taken from the center of each defect were stained with either safranin O and fast green or picrosirius red and imaged at ×10 under brightfield using a Zeiss Axio Scan.Z1 slide scanner. Picrosirius red–stained slides were also imaged under polarized light using a Zeiss Axio Scan.Z1 slide scanner. Safranin O/fast green images were cropped to only show empty or autograft repair tissue (see Figure 5, A and B) and were scored by 4 blinded observers (B.D.S., R.A.F., E.R.B., L.G.L.) using the OARSI histopathology scoring system.^34^ The mean of the independent scores of the 4 observers was computed for each slide for each parameter.

### Statistical Analysis

Descriptive statistics were computed using Microsoft Excel. All other statistical analyses were performed using GraphPad Prism Version 10. Statistical outliers were detected and excluded using the ROUT method with a *Q* value of 1%. Comparisons between testing sites (autograft center and adjacent, empty defect center and adjacent, and contralateral) for mechanical testing parameters were computed using a 2-way analysis of variance separately for untreated and Anakinra-treated cohorts. Post hoc Tukey tests were used for pairwise comparisons where significant effects were found (α = .05). A Student *t* test (2-tailed, unpaired) was used to determine differences between untreated and Anakinra-treated specimens or between operative and contralateral limbs within each treatment group, with statistical significance set at a *P* value ≤.05 for all other quantitative results.

## Results

### Surgical Outcomes

One subject in the Anakinra-treated group was eliminated from further cartilage analysis due to cartilage-bone delamination of the graft that occurred during surgery. Otherwise, no other complications occurred. All animals in this study recovered uneventfully from surgery, with pain managed well by the standard course of analgesics provided postsurgery, and reached their intended time points 5 weeks after surgery.

### Synovial Histopathology

**Figure 2.**
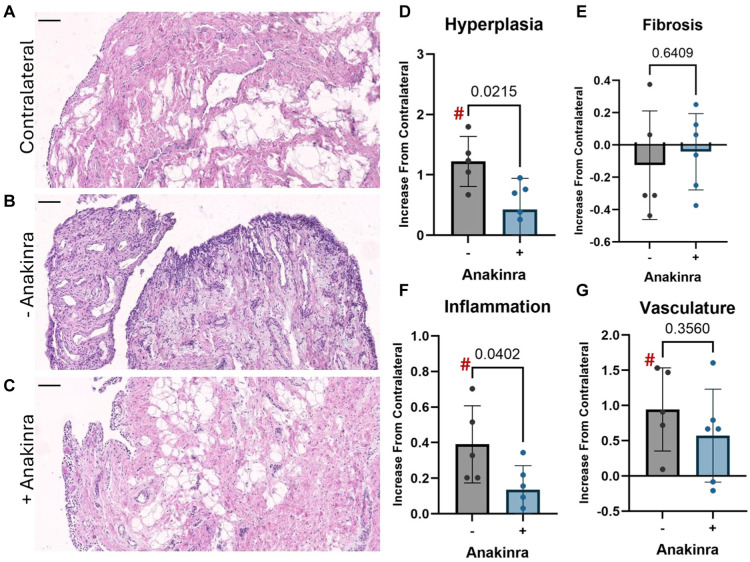
(A-C) Representative hematoxylin and eosin–stained synovium sections from contralateral stifle joints, operated stifles without Anakinra treatment, and operated stifles with weekly intra-articular Anakinra. Scale bar = 100 µm. (D-G) Synovial histopathology scores for lining hyperplasia (D), subintimal fibrosis (E), inflammation (F), and vasculature (G) in operative stifles 5 weeks after surgery, expressed as an increase relative to the contralateral baseline. **#** = statistically significant difference from contralateral baseline; +/– = with or without IL1-ra administration.

Synovium sections were stained with hematoxylin and eosin to visualize cell and tissue morphology (Figure 2, A-C) and scored for synovial pathology (inflammation, hyperplasia, vasculature, and fibrosis). Scores were averaged among graders and fields of view yielding a single score per parameter for each specimen. Interrater reliability was determined to be between moderate and strong agreement among graders (see Supplemental Table 1). Scores are presented as change from contralateral, computed by subtracting the mean score for the contralateral stifles from each experimental animal (untreated or Anakinra-treated) (Figure 2, D-G). In untreated animals, the score for lining hyperplasia was significantly higher in operative stifles compared with contralateral (*P* = .0022), and while elevated, this increase did not reach statistical significance in Anakinra-treated subjects (*P* = .119). The increase from contralateral was significantly (*P* = .022) greater without Anakinra treatment (Figure 2D). The fibrosis parameter was unchanged from contralateral in both groups (Figure 2E). Synovium from operative stifles had significantly more inflammatory cell infiltration compared with contralateral in untreated animals (*P* = .011). While this metric was again elevated, the increase did not reach statistical significance with Anakinra treatment (*P* = .058). The increase from contralateral was significantly greater (*P* = .04) in untreated stifles compared with treated (Figure 2F). Similarly, the vascular score significantly increased in the operative stifle compared with contralateral in untreated joints (*P* = .028), while the increase was not statistically significant in Anakinra-treated joints (*P* = .088). However, when comparing the change from contralateral between Anakinra-treated and untreated joints, there was no statistically significant difference (*P* = .36) between groups (Figure 2G).

### Cartilage Mechanics

**Figure 3.**
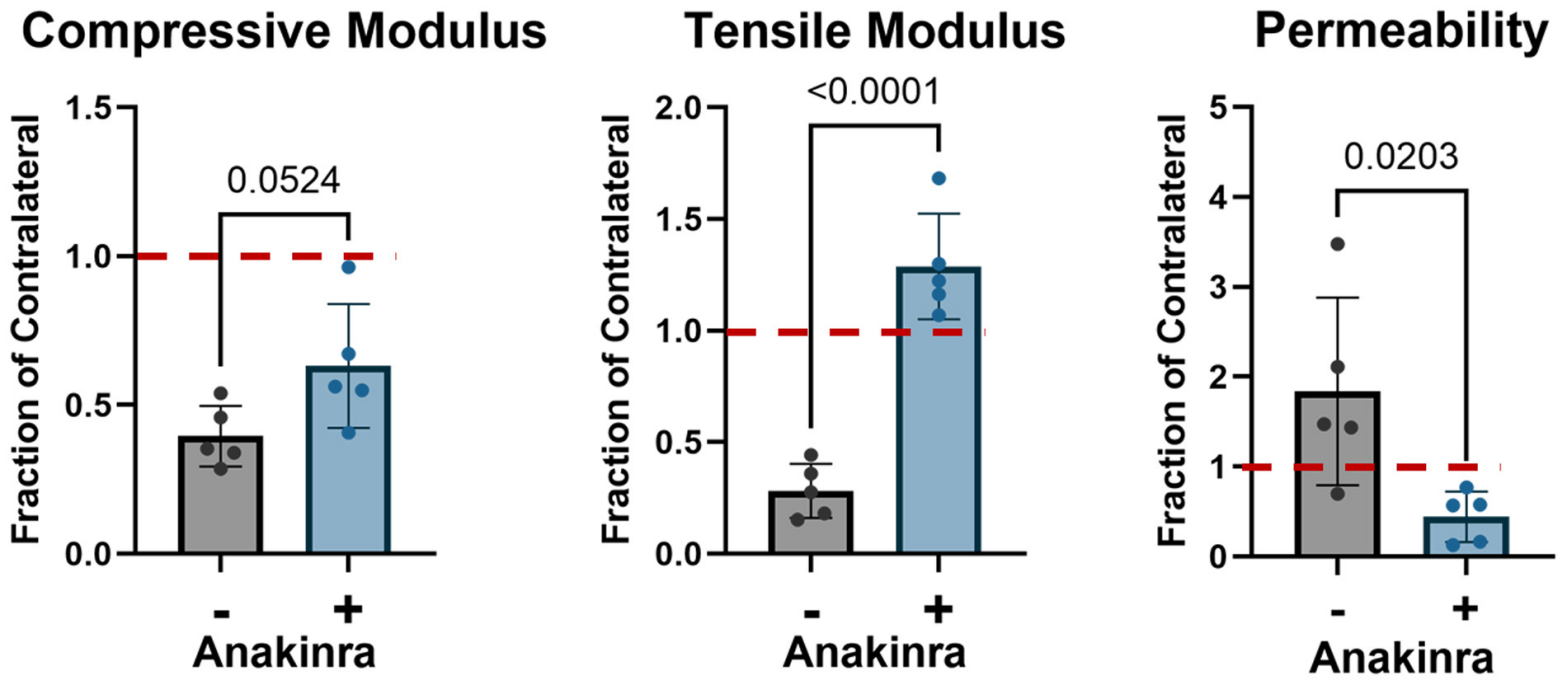
Cartilage mechanical properties in the center of each osteochondral autograft plug 5 weeks after surgical implantation, expressed as a fraction of the contralateral stifle (dashed line). +/– = with or without IL1-ra administration.

The material parameters of compressive modulus (*E_y_*_–_), tensile modulus (*E_y_*_+_), and zero-strain permeability (*k*_0_) were computed for each testing location by fitting the indentation creep deformation curve to a Hertzian biphasic analytical model.^40^ The contralateral means of these parameters for each treatment group were computed and used to normalize results in the operative stifle as the fraction of contralateral. The compressive modulus of the autograft cartilage in untreated stifles was 39.5% ± 10.2% of contralateral, while in Anakinra-treated stifles it was 63.1% ± 20.8% of contralateral. The tensile modulus of the autograft cartilage in untreated stifles was 28.2% ± 12.1% of contralateral cartilage—significantly less (*P* < .0001) than in Anakinra-treated stifles (128.8% ± 23.6% of contralateral). Zero-strain permeability in untreated stifles was 183.9% ± 104.4% of contralateral—significantly elevated (*P* = .02) in comparison with that of Anakinra-treated stifles (44.2% ± 28.2% of contralateral) (Figure 3).

In terms of absolute values before normalization, in the absence of Anakinra treatment, autograft cartilage had significantly lower (*P* = .0031) compressive modulus than contralateral cartilage (0.49 ± 0.13 MPa vs 1.25 ± 0.33 MPa). Similarly, tensile modulus was significantly reduced (*P* = .0085, 3.59 ± 1.54 MPa vs 12.73 ± 5.43 MPa). Permeability did not show a significant change (0.0081 ± 0.0046 mm^4^/Ns vs 0.0054 ± 0.0054 mm^4^/Ns). With anakinra treatment, only compressive modulus was significantly reduced (*P* = .029) in autograft cartilage in comparison with contralateral (0.93 ± 0.31 MPa vs 1.48 ± 0.35 MPa). Tensile modulus and permeability were unchanged compared to contralateral with Anakinra treatment (Supplemental Figure S1).

Unsurprisingly, the tissue filling the empty defects at 5 weeks was significantly softer and more permeable across all parameters and treatment groups in comparison with the corresponding contralateral cartilage (Supplemental Figure S1). However, when comparing empty defect tissue between untreated and Anakinra-treated stifles as a fraction of the contralateral, infill tissue in Anakinra-treated stifles was significantly stiffer than untreated (*P* = .0015; 35.9% ± 16.1% vs 3.1% ± 1.7%) (Supplemental Figure S2).

### Osteochondral Histology

**Figure 4.**
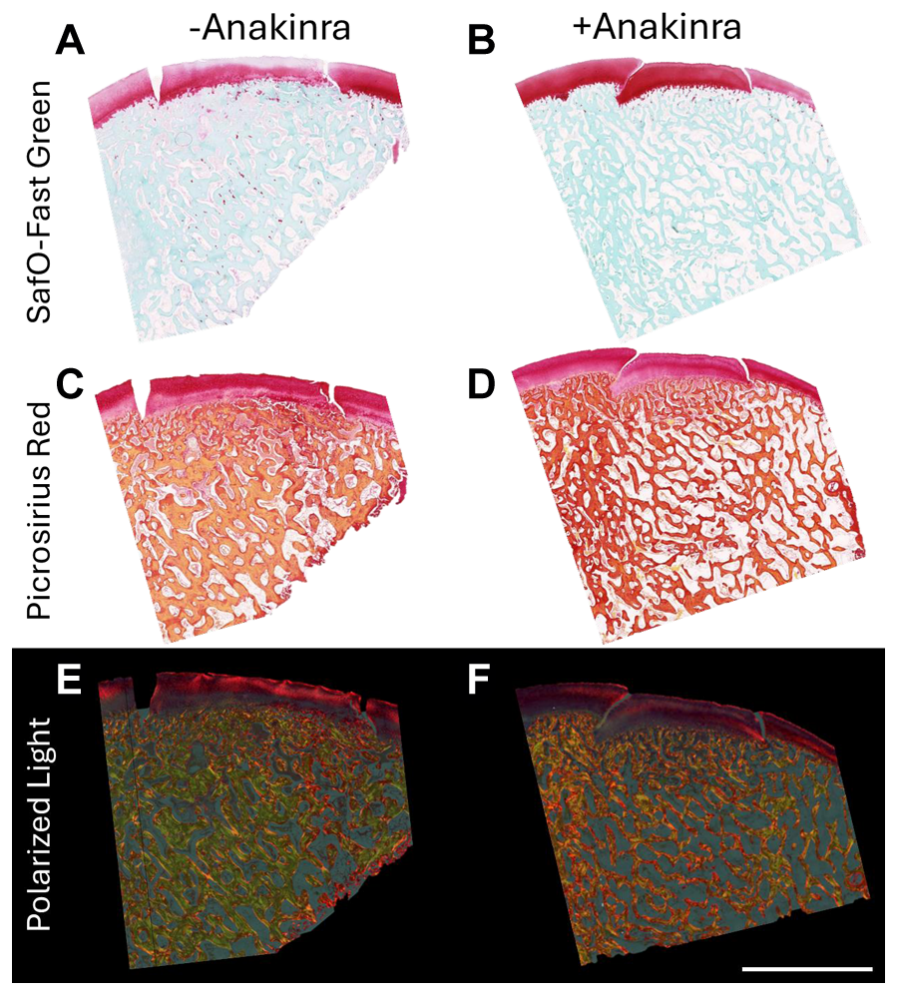
(A and B) Representative safranin O– and fast green–stained medial femoral condyle sections from untreated and Anakinra-treated stifles at the midplane of each autograft. (C and D) Picrosirius red–stained medial femoral condyle sections imaged under brightfield. (E and F) Picrosirius red–stained medial femoral condyle sections imaged under polarized light. Scale bar = 5 mm.

Sagittal sections through the autograft and empty defects on each medial femoral condyle were stained with safranin O and fast green to visualize ECM and tissue structure (Figure 4, A and B; Supplemental Figure S5). Qualitatively, regardless of treatment group, bony integration of autografts was excellent, with few if any discontinuities along the bony margin. However, graft edges were still plainly visible in the cartilage region, with some loss of proteoglycan content and mild surface roughening (especially in untreated stifles). Picrosirius red staining showed collagen content and structure (Figure 4, C and D), which again exhibited tissue discontinuity in the cartilage layer between the autograft and adjacent cartilage. When imaged with polarized light (Figure 4, E and F), which allows for the visualization of collagen architecture and organization, it was evident that the tissue was more disorganized toward the defect edges, as well as across the superficial layer of the autograft cartilage. This disorganization appeared to progress deeper into the cartilage tissue in untreated specimens compared to those treated with Anakinra. As expected, empty defects healed poorly at this time point, with the defect fill consisting largely of disorganized, collagen-rich fibrous tissue and little to no proteoglycan content. Notably, however, new bone deposition appeared to have been accelerated in empty defects with Anakinra treatment (Supplemental Figure S3).

**Figure 5.**
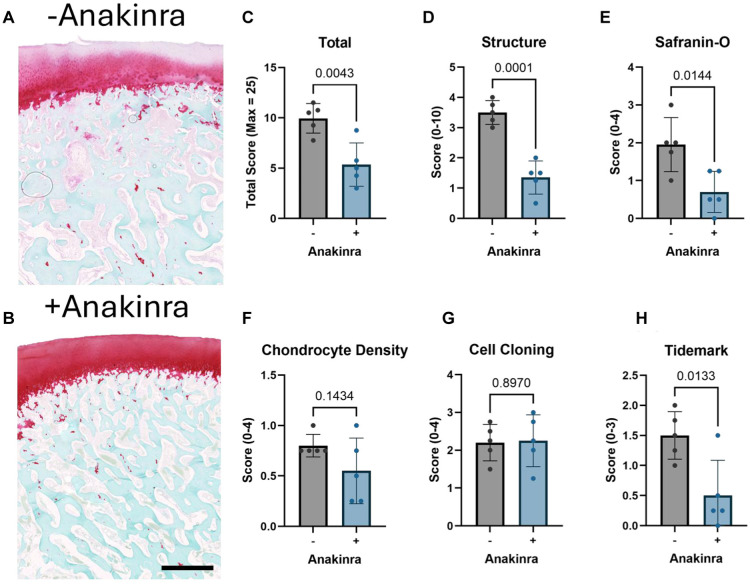
(A and B) Representative safranin O– and fast green–stained medial femoral condyle sections from untreated and Anakinra-treated stifles cropped and zoomed for scoring of the center of each autograft. Scale bar = 1 mm. (C) Total Osteoarthritis Research Society International histopathology score. (D-H) Sum of subscores for structure, safranin O staining intensity, chondrocyte density, cell cloning, and tidemark. +/– = with or without IL1-ra administration.

Safranin O– and fast green–stained images were cropped to show only the center of each autograft for scoring using the OARSI histopathology scoring system^34^ by 4 trained and blinded observers (B.D.S., R.A.F., E.R.B., L.G.L.) (Figure 5, A and B). Interrater reliability was determined to be between strong and perfect agreement among graders (see Supplemental Table 1). This analysis yielded scores for 5 parameters: structure, interterritorial safranin O, chondrocyte density, cell cloning, and tidemark. The sum of these parameters was computed as the total histopathology score for each specimen, with higher numbers corresponding to a greater degree of degeneration. The total score was significantly higher (indicating more degeneration) for autografts in untreated stifles in comparison to those in Anakinra-treated joints (*P* = .0043; 9.95 ± 1.47 vs 5.35 ± 2.15, out of a maximum score of 25) (Figure 5C). The difference was readily apparent in the structure subscore (*P* = .0001; 3.50 ± 0.40 vs 1.35 ± 0.55, out of 10) (Figure 5D) and in the safranin O staining intensity score (*P* = .014; 1.95 ± 0.72 vs 0.70 ± 0.54, out of 4) (Figure 5E), as well as in the tidemark assessment subscore (*P* = .013; 1.50 ± 0.40 vs 0.50 ± 0.59, out of 3) (Figure 5H). There were no significant differences in either chondrocyte density (Figure 5F) or cell cloning (Figure 5G) subscores.

### Micro-Computed Tomography

**Figure 6.**
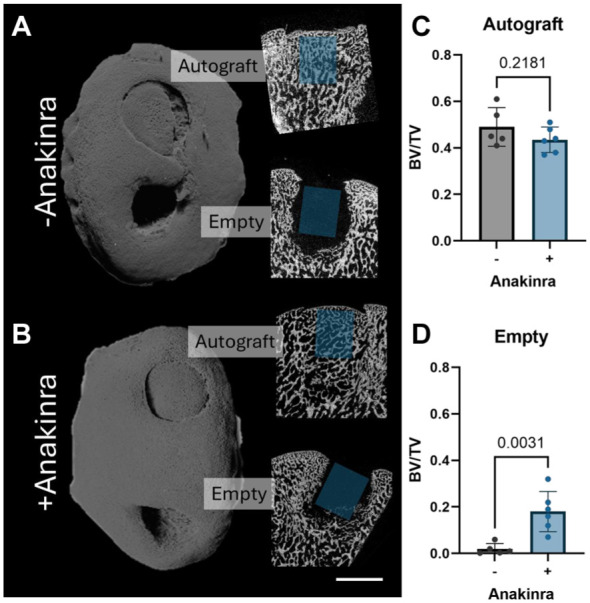
(A and B) Representative 3-dimensional renderings of the medial femoral condyle and sagittal 2-dimensional cross sections of autografts and empty defects for untreated and Anakinra-treated stifles. Scale bar = 5 mm. (C and D) Bone volume fraction within a cylindrical region of interest 4 mm in diameter and 5 mm in height beginning approximately 1 mm below the bone-cartilage interface in the center of each autograft and empty defect. BV, bone volume; TV, total volume; +/– = with or without IL1-ra administration.

Three-dimensional microCT reconstructions of the operative medial femoral condyles showed excellent autograft retention and shape conformation, regardless of treatment group (Figure 6, A and B). Similarly, 2-dimensional slices across the center of each autograft demonstrated fully integrated osseus tissue at this 5-week time point, both with and without Anakinra treatment. This was reflected in the calculation of bone volume fraction within the autograft (*P* = .22; 0.49 ± 0.083 for untreated vs 0.44 ± 0.055 for Anakinra treated) (Figure 6C). There appeared to be more new bone formation in the empty defects of Anakinra-treated stifles, which was confirmed via calculation of the bone volume fraction (*P* = .0031; 0.019 ± 0.02 for untreated vs 0.18 ± 0.09 for Anakinra treated) (Figure 6D).

## Discussion

The goal of this study was to determine whether an adjuvant therapy, in this case intra-articular injection of recombinant IL1-ra (Anakinra), after an OAT procedure could enhance repair and ameliorate degenerative changes to graft tissue and improve overall joint health after surgery. We hypothesized that by blocking IL-1β signaling in the postoperative period, the deleterious effects of joint inflammation on healing could be attenuated. Our results show that, at an early 5-week time point, synovial pathology was significantly reduced in operative stifles receiving IL1-ra treatment in comparison with untreated controls. This is consistent with prior work from our group that showed that synovial explants from client-owned canines presenting with cruciate ligament injury displayed numerous histological and gene expression signatures of inflammatory pathology, but when these explants were treated with IL-1ra in culture, this inflammatory phenotype was significantly attenuated.^33^

Synovial pathology, and the concurrent joint-wide inflammation that it implies, is a major signature of the degenerative changes that presage OA^50^ and may contribute directly to cartilage degradation through MMP-mediated catabolism.^26,55^ While MMPs play a role in normal cartilage tissue homeostasis, the upregulation that accompanies joint inflammation results in catabolic matrix degradation in the cartilage, and IL-1β (as well as other inflammatory cytokines) is a major contributor to the overexpression of MMP enzymes in injured and inflamed joints.^26,55^ In vitro experiments clearly demonstrate the negative effects of IL-1β on the ECM content, structure, and integration potential of cartilage tissue,^12,39^ which may be rescued with IL-1ra treatment.^48^

This may help explain some of the most striking results in the current study. First, indentation creep testing showed that autografts better maintained their functional integrity in IL-1ra–treated operative stifles compared with untreated controls. This result was especially noteworthy in the parameters of tensile modulus and permeability, the former of which is directly related to the integrity of the collagen network in the superficial zone of cartilage.^53^ Evaluation of the osteochondral histology, both qualitatively and quantitatively (via the structure OARSI subscore), clearly indicates that superficial zone structural perturbations are more pronounced in the autografts of untreated joints. Furthermore, with IL-1ra treatment, proteoglycan content (measured through safranin O staining) was better maintained. This combination implies a collagen matrix–preserving (or ECM deposition–promoting) effect of IL-1ra treatment after osteochondral autografting. This finding is further evidenced by analysis of the tissue ingrowth into the empty donor site defects. Picrosirius red–stained histological sections showed highly collagenous fibrous tissue infill in empty defects, and while still far below the contralateral baseline, mechanical testing revealed that this tissue was significantly stiffer in IL-1ra–treated joints in comparison with untreated. A recent study from our group in the context of annulus fibrosis repair showed that fibrous scar formation at the injury site was significantly greater with local IL-1ra release.^47^ However, this direct or indirect influence on collagen network turnover leading to growth of fibrous tissue may not always be desirable, especially in the context of cartilage repair, and the potential negative consequences of this phenomenon will require careful study.

The same inflammatory cytokines that spur cartilage degradation may also adversely impact bone healing.^27,30^ Rapid osseus integration is vital to the survival of osteochondral grafts,^9^ and abnormal bone healing, including subchondral and basal cyst formation and communication between the joint space and underlying bone, has been implicated in osteochondral graft failure.^46^ Inadequate closure of the subchondral plate has been implicated as a major driver in donor site morbidity in osteochondral autograft procedures.^42^ Incomplete lateral integration between mating cartilage surfaces may allow communication between the joint space (and its cytokine-containing synovial fluid) and the subchondral bone, which may be a mechanism of bone cyst formation.^46^ We did not observe any evidence of subchondral or basal bone cysts in any of our specimens, which is perhaps unsurprising given the early time point. However, in the autografts of our untreated control group we did observe histological evidence of abnormalities in the subchondral bone plate as shown by the elevated tidemark score. Interestingly, this was significantly improved in our Anakinra-treated group. There is evidence in the literature that IL-1ra may enhance bone healing. For instance, Lackington et al^31,32^ showed that IL1-ra delivery rescues the osteogenic potential of mesenchymal stromal cells cultured in the presence of IL-1β and, in a follow-up in vivo study, showed that IL-1ra enhanced the efficacy of recombinant human bone morphogenic protein–2 in a femoral defect model of bone healing in a rat. Our data support the hypothesis that IL-1ra enhances bone healing. As evidenced by our microCT results, the empty defects from Anakinra-treated stifle joints show significantly more bony deposition than those of untreated controls, which may improve and accelerate osteointegration of the autografts. Additional early time points, coupled with quantitative assessment of bone formation rates,^63,64^ will be required to assess this impact of IL-1ra on the rate of autograft bony integration.

Several prior studies have investigated adjuvant therapeutics applied either during surgery or in the immediate postoperative period in the context of osteochondral repair in large animal models. Tibesku et al^57^ found that intra-articular administration of hepatocyte growth factor after an OAT procedure in sheep produced less histological evidence of abnormal cell cloning in the graft cartilage compared with untreated control knees, with more subtle improvements in other histological outcomes. Siebert et al^54^ performed an OAT procedure on the femoral condyles of sheep, with experimental grafts bathed in either basic fibroblast growth factor or bone morphogenic protein–2 before reimplantation, and found accelerated osseus integration with either growth factor in comparison to controls, but no differences in cartilage healing. Stefani et al^56^ implanted an acellular dexamethasone-eluting graft into the autograft donor site during an OAT procedure in a canine model and observed improved functional outcomes and osteochondral healing but no differences in synovial fluid cytokine content compared with controls. They postulated the main effect of treatment to be pro-anabolic as opposed to anti-inflammatory.

Several limitations of this study will inform future directions. While we have shown promising results in the potential of intra-articular IL-1ra to enhance graft function in an in vivo model of osteochondral repair, we have not optimized the dosing regimen. Anakinra has a relatively short half-life systemically,^26^ and the pharmacokinetics of this molecule in the joint environment is not fully understood. Furthermore, we do not know the durability of repair, and whether the protective effect of treatment on graft integrity is transient, necessitating longer endpoints in future studies. Should this be the case, we could consider inclusion of IL-1ra in drug delivery vehicles to prolong joint residence time.^47,48,63^ Although these data support several hypotheses related to the mechanism of action leading to the marked improvements in graft function that we observed with IL-1ra treatment (reduction of inflammatory cytokine-driven catabolism and improved bone integration), experiments to specifically test these hypotheses in the appropriate time window will be required. Moreover, longer-term endpoints will be necessary to evaluate functional and behavioral outcomes such as pain, gait, and general activity, and differences between treatment groups may not become measurable until later time points. Future studies will also be required to evaluate and monitor off-target effects of intra-articular Anakinra treatment. Finally, although our intra-articular injection volume was small (1 mL), and we do not expect a response from the injection itself, we did not compare either of our experimental groups to saline carrier control. With regard to clinical relevance, there are several additional limitations to this study. Unlike humans, animals were allowed unrestricted weightbearing as tolerated immediately after surgery. Despite this, all implants remained well in place in both study cohorts; however, this early resumption of load-bearing activity may have influenced graft outcomes.

The results of this study show that adjuvant anti-inflammatory therapeutics delivered directly to a knee joint after an osteochondral repair procedure have the potential to enhance graft integrity and improve repair outcomes. This may not only enhance the results of osteochondral repair procedures as they are performed currently but may also expand indications for such treatments to a greater population of patients. OAT (and indeed all biological repair techniques) is currently only performed in near-ideal surgical scenarios, and patients with other comorbidities (including inflammatory conditions and OA) are nearly always excluded.^37^ This “red knee” population, which represents the vast majority of patients receiving care for knee joint injury and degeneration, is currently treated palliatively, and many will eventually require a total knee replacement.^37^ Adjuvant therapeutics such as the one described in this study may allow these patients to become candidates for the most advanced biological repair strategies, from which they are currently excluded.

## Supplemental Material

sj-docx-1-ajs-10.1177_03635465251401225 – Supplemental material for Intra-articular Delivery of Recombinant Interleukin-1 Receptor Antagonist Protein (Anakinra) Enhances Graft Function in a Porcine Model of Osteochondral Repair Supplemental material, sj-docx-1-ajs-10.1177_03635465251401225 for Intra-articular Delivery of Recombinant Interleukin-1 Receptor Antagonist Protein (Anakinra) Enhances Graft Function in a Porcine Model of Osteochondral Repair by Brendan D. Stoeckl, Rachel A. Flaugh, Akbar N. Syed, Kendall M. Masada, Elizabeth R. Bernstein, Elisabeth A. Lemmon, Austin C. Jenk, Lorielle G. Laforest, Natalie L. Fogarty, Bijan Dehghani, Carla R. Scanzello, James L. Carey, David R. Steinberg and Robert L. Mauck in The American Journal of Sports Medicine
