# Supplementary material for: Intra-articular Delivery of Recombinant Interleukin-1 Receptor Antagonist Protein (Anakinra) Enhances Graft Function in a Porcine Model of Osteochondral Repair
Source: Am J Sports Med. 2026 Jan 21;54(3):647–57. doi: 10.1177/03635465251401225 (PMC12916876; doi:10.1177/03635465251401225)
Supplement: sj-docx-1-ajs-10.1177_03635465251401225 – Supplemental material for Intra-articular Delivery of Recombinant Interleukin-1 Receptor Antagonist Protein (Anakinra) Enhances Graft Function in a Porcine Model of Osteochondral Repair [file sj-docx-1-ajs-10.1177_03635465251401225.docx]

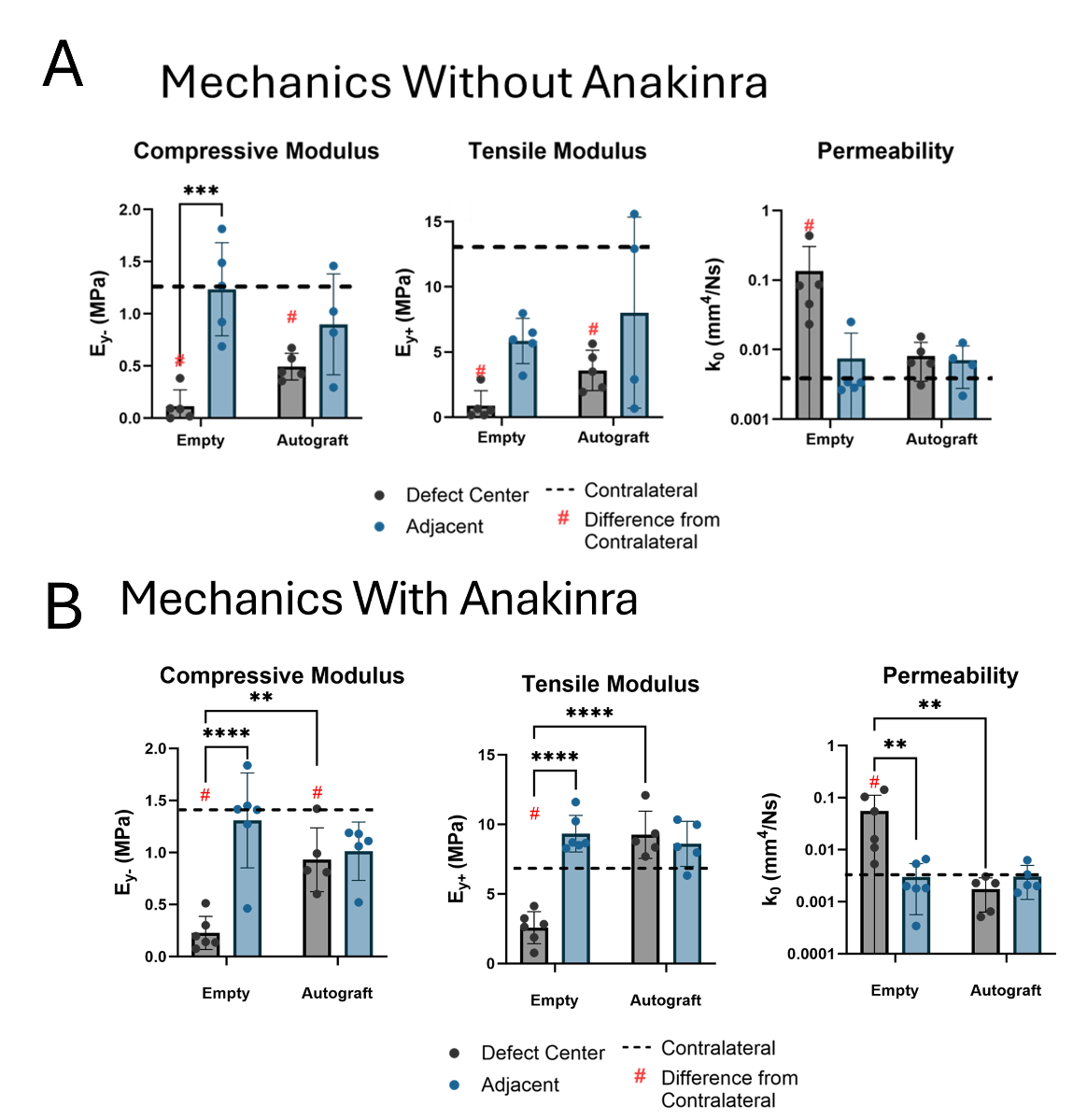


**Supplemental Figure S1:** Full set of mechanical parameters from indentation creep testing both in the center of and 5 mm adjacent to each autograft or empty defect for **A)** untreated and **B)** Anakinra treated stifle joints.


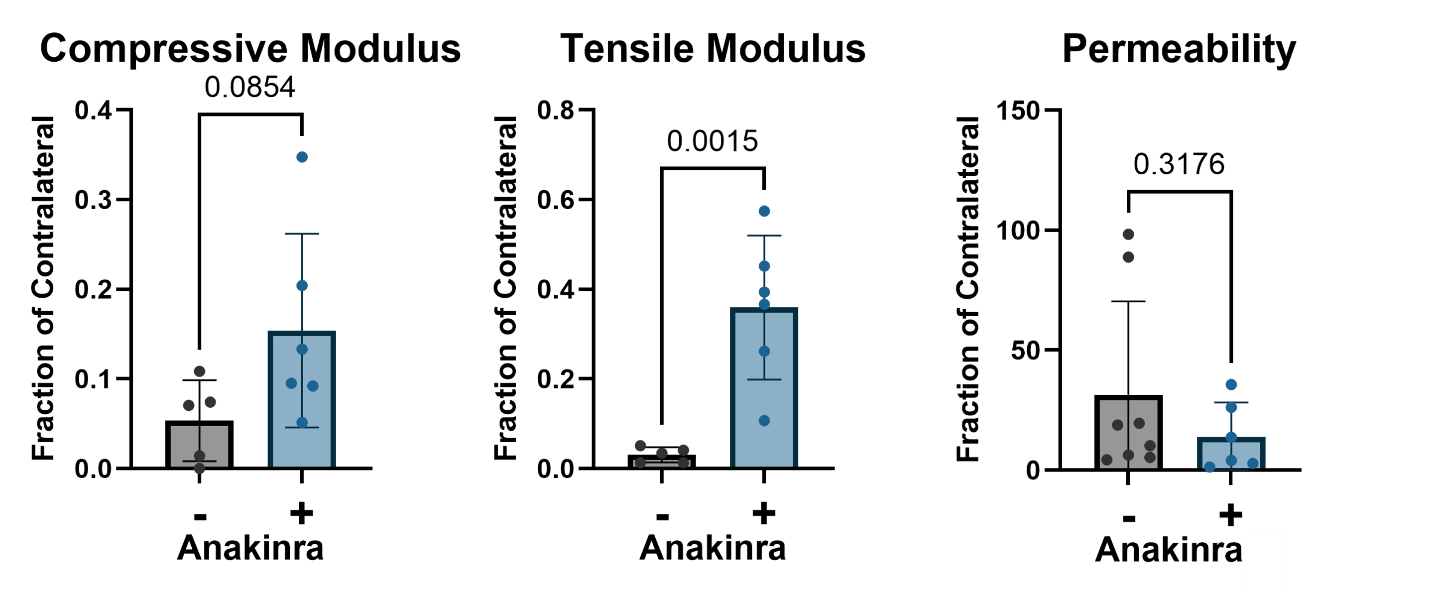


**Supplemental Figure S2:** Cartilage mechanical parameters from creep indentation testing in the center of each empty defect five weeks after surgical implantation, expressed as a fraction of the of the contralateral stifle. +/- indicates with or without IL-1ra treatment.


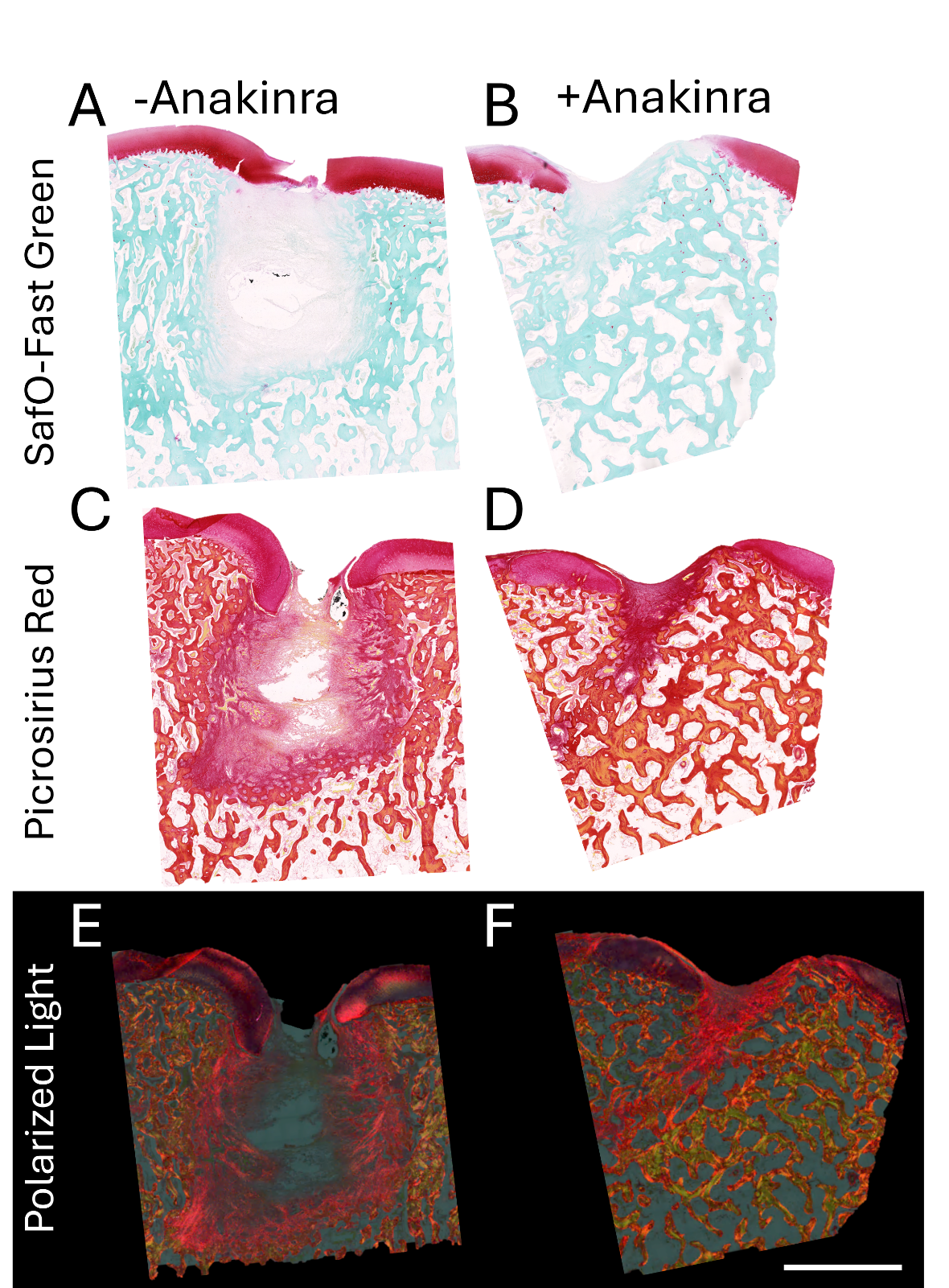


**Supplemental Figure S3: A-B)** Representative Safranin O- and Fast Green-stained medial femoral condyle sections from untreated and Anakinra treated stifles at the midplane of each empty defect. **C-D)** Picrosirius Red stained medial femoral condyle sections imaged under brightfield. **E-F)** Picrosirius Red stained medial femoral condyle sections imaged under polarized light. Scale = 5 mm.


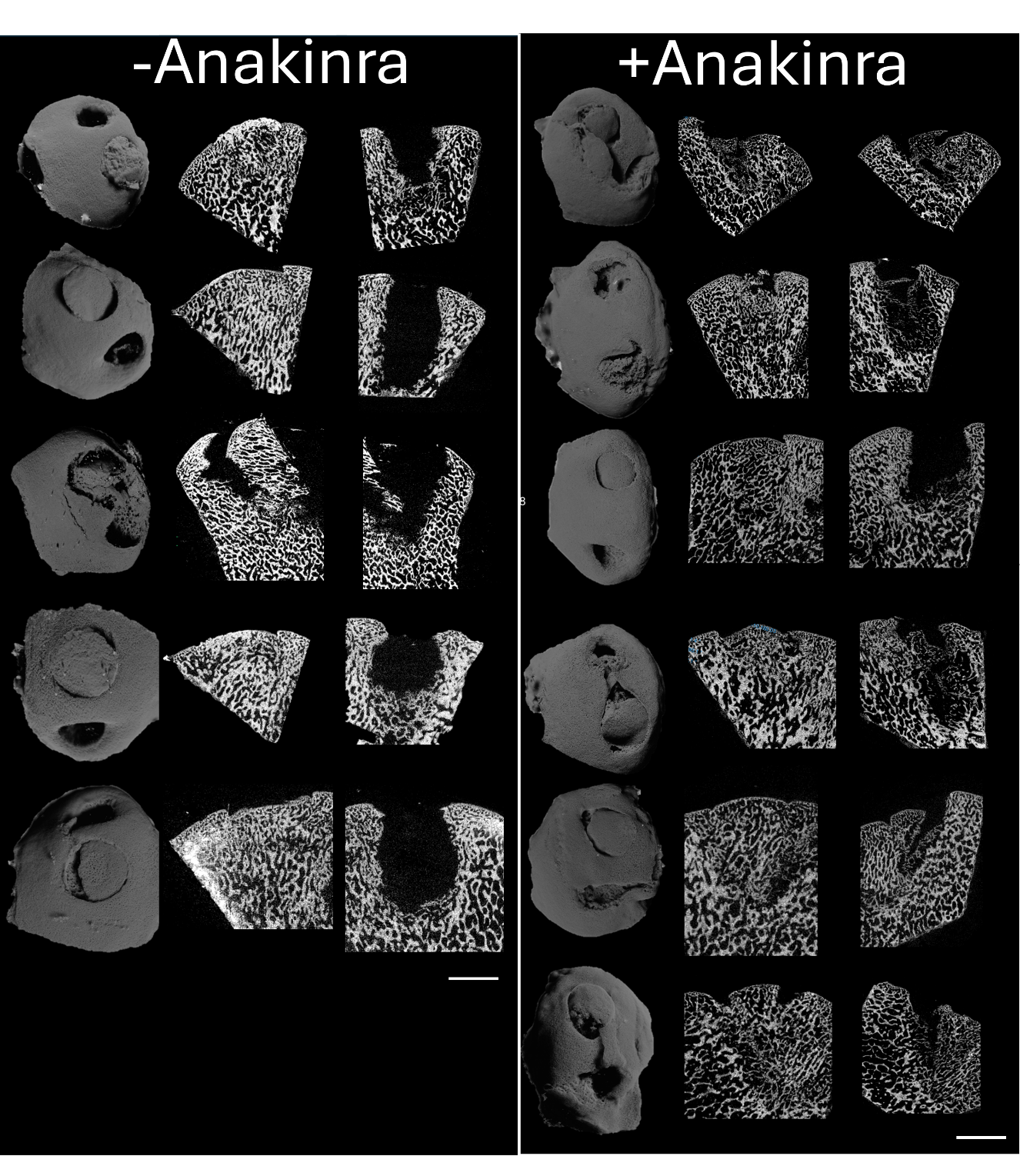


**Supplemental Figure S4:** 3D renderings of the medial femoral condyle and sagittal 2-D cross-sections of autografts and empty defects for all untreated and Anakinra-treated stifles. Scale =5 mm.


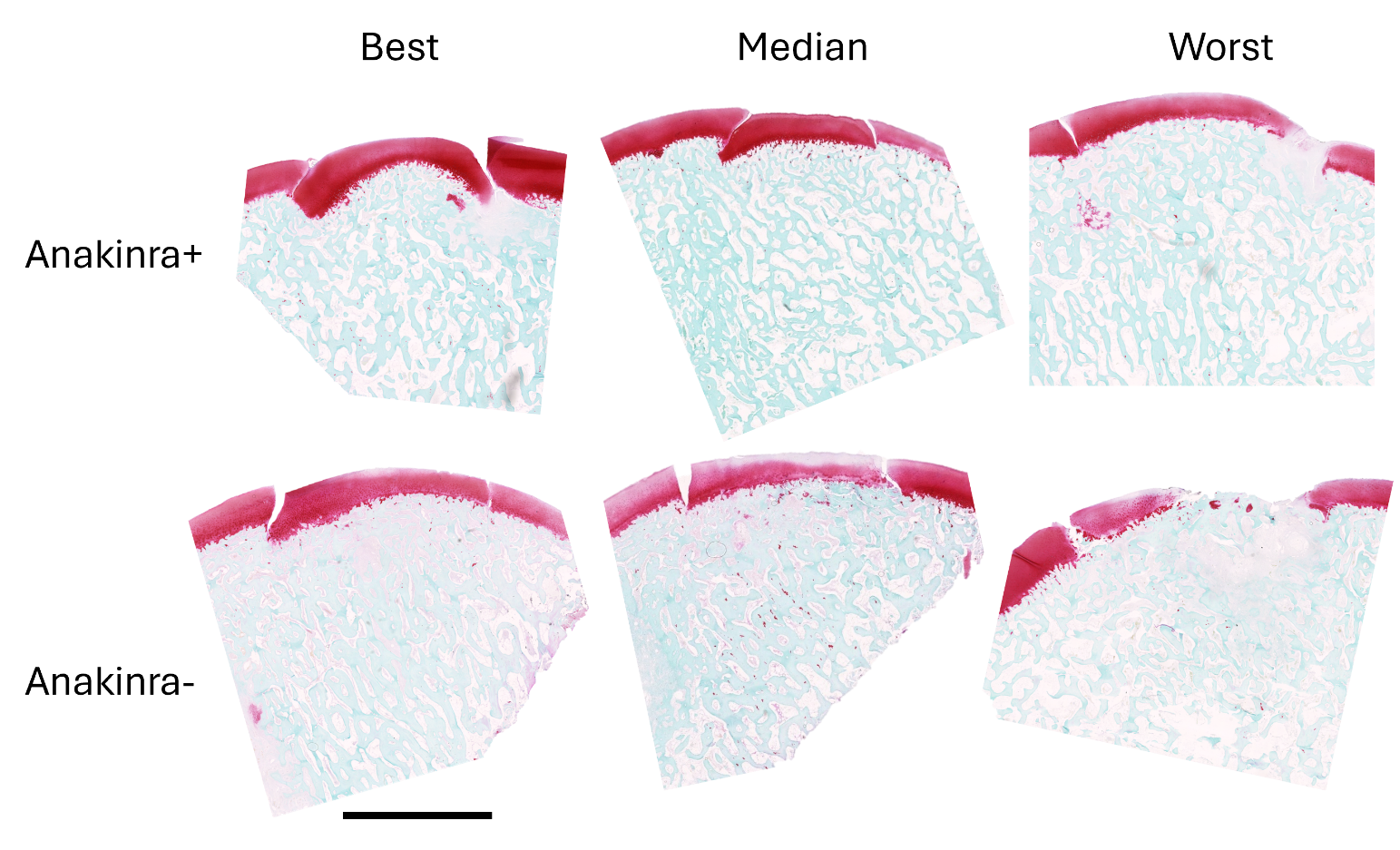


**Supplemental Figure S5:** Best, median and worst safranin-O Fast green stained medial femoral condyle sections from untreated and Anakinra treated stifles near the midplane of each autograft. Scale = 5mm.

**Osteochondral:**

| **Kendall's Coefficient of Concordance W** | | |
| --- | --- | --- |
| Structure | 0.7002167 | Strong Agreement |
| Chondrocyte Density | 0.9022511 | Perfect Agreement |
| Cell Cloning | 0.8317134 | Strong Agreement |
| Safranin O | 0.9270164 | Perfect Agreement |
| Tidemark | 0.8780673 | Strong Agreement |

**Synovium**:

| **Kendall's Coefficient of Concordance W** | | |
| --- | --- | --- |
| Hyperplasia | 0.8460261 | Strong Agreement |
| Vasculature | 0.7775572 | Strong Agreement |
| Inflammation | 0.5650911 | Moderate Agreement |
| Fibrosis | 0.5431326 | Moderate Agreement |

**Supplemental Table 1:** Inter-rater reliability calculations for histopathology scoring.
